# Immunization errors of COVID-19 vaccines in Minas Gerais

**DOI:** 10.1590/0034-7167-2023-0525

**Published:** 2025-08-08

**Authors:** Wiara Viana Ferreira, Thayane Ingrid Xavier de Andrade, Thays Cristina Pereira Barbosa, Rillary Carvalho dos Santos, Stênio Henrique Oliveira, Vinícius Carvalho Guimarães, Eliete Albano de Azevedo Guimarães, Valeria Conceição de Oliveira

**Affiliations:** I Universidade Federal de São João Del Rei. Divinópolis, Minas Gerais, Brazil; II Universidade de São Paulo. Ribeirão Preto, São Paulo, Brazil

**Keywords:** COVID-19 Vaccines, Medication Errors, Immunization Programs, Mass Vaccination, Epidemiology Descriptive., Vacunas contra la COVID-19, Errores de Medicación, Programas de Inmunización, Vacunación Masiva, Epidemiología Descriptiva.

## Abstract

**Objectives:**

to analyze immunization errors related to COVID-19 vaccines in Minas Gerais.

**Methods:**

a descriptive epidemiological study, carried out in Minas Gerais with data from e-SUS Notifica in 2021. Descriptive analysis and calculation of the incidence rate by health macro-regions of the state were performed.

**Results:**

of the 853 municipalities, only 381 (44.7%) reported some immunization error, totaling 2,724 reports. The incidence of errors reported in the state was 7.8/100 thousand doses applied, with the Vale do Aço macro-region (14.4/100 thousand) having the highest incidence. The most frequent error was the type of vaccine used (37.3%), and the Pfizer vaccine had the lowest incidence of error.

**Conclusions:**

the COVID-19 vaccine immunization errors reported in Minas Gerais point to the heterogeneity in incidence rates in the various macro-regions of the state, in addition to the underreporting of these errors.

## RESUMEN

**Objetivos:**

analizar errores de inmunización relacionados con las vacunas contra COVID-19 en Minas Gerais.

**Métodos:**

estudio epidemiológico descriptivo, realizado en Minas Gerais con datos de e-SUS Notifica en 2021. Se realizó análisis descriptivo y cálculo de la tasa de incidencia por macrorregiones sanitarias del estado.

**Resultados:**

de los 853 municipios, sólo 381 (44,7%) reportaron error de vacunación, totalizando 2.724 notificaciones. La incidencia de errores reportados en el estado fue de 7,8/100 mil dosis aplicadas, siendo la macrorregión de Vale do Aço (14,4/100 mil) la de mayor incidencia. El error más frecuente fue el tipo de vacuna utilizada (37,3%), y la vacuna Pfizer tuvo la menor incidencia de error.

**Conclusiones:**

los errores de inmunización con la vacuna COVID-19 reportados en Minas Gerais apuntan a la heterogeneidad en las tasas de incidencia en las distintas macrorregiones del estado, además del subregistro de estos errores.

## INTRODUCTION

In March 2020, the World Health Organization declared the outbreak of the new coronavirus, SARS-CoV-2, a pandemic^(1)^, and throughout that year, the population closely followed the efforts of scientific community in developing the stages of studies of vaccines against SARS-CoV-2^(2)^. On January 17, 2021, the Brazilian National Health Regulatory Agency (In Portuguese, *Agência Nacional de Vigilância Sanitária* - ANVISA) granted authorization for the emergency use of Sinovac/Butantan COVID-19 (inactivated) adsorbed vaccine and AstraZeneca/Fiocruz COVID-19 (recombinant) vaccine^(2)^.

To coordinate the vaccination campaign in Brazil, the Brazilian National Plan for the Operationalization of COVID-19 vaccination was created with the aim of structuring the distribution of vaccines and defining priority groups^(3)^. During 2021, the vaccines approved and introduced were CoronaVac/Butantan COVID-19 (inactivated) adsorbed vaccine, AstraZeneca COVID-19 (recombinant) vaccine, Comirnaty Pfizer COVID-19 (mRNA) vaccine and Janssen COVID-19 (recombinant) vaccine, which allowed expanding vaccination beyond priority groups and accelerating the population vaccination process^(3,4)^.

Given the need to expand and speed up vaccination against COVID-19, several strategies were implemented, such as the creation of drive-thrus, the hiring of more healthcare professionals to administer vaccines, the continuous training of these professionals and the offering of home vaccinations^(5,6)^. These measures were intended to ensure that the greatest number of people were vaccinated quickly and efficiently, but they created potential risks to patient safety due to the possibility of immunization errors (IE).

IEs are not simply the result of individual failures, but the result of interactions between individuals and the systems in which they are embedded^(7)^, often being the product of a complex dynamic between different factors. These factors may include organizational culture, systems and processes, lack of resources, inadequate communication, job stress, insufficient training, among others^(8)^. Errors are inherent in any human action, regardless of the field of activity, reflecting a constant characteristic of the human cognitive process^(7)^.

IE can be defined as any preventable event due to the incorrect use of immunobiological agents, procedures and systems that may cause a reduction or absence of immunity and a serious event supposedly attributable to vaccination or immunization (ESAVI)^(9)^.

When we analyze all the factors that can lead to IE, we realize that the professional who triggered it is at the final stage of the chain, but may not be the origin of the problem. If it is possible to intervene at this stage, through preventive actions, it is possible to avoid future mistakes^(7)^.

Nursing staff act as a final barrier in preventing IE^(10)^. However, the vaccination process is complex and involves several steps, including production, distribution logistics, storage, management, and communication, each of which is essential to successful vaccination. Taking shared responsibility for patient safety is crucial to developing and maintaining safe and efficient health systems^(11)^.

The literature has already indicated that the introduction of new immunobiological agents is a contributing factor to the increase in IE^(12-15)^. Furthermore, in large-scale situations, such as the vaccination campaign against COVID-19, an increase in the incidence of errors and ESAVI may occur^(16,17)^. Therefore, it is important to monitor the occurrence of errors to understand and elucidate their causes and effects, in addition to implementing preventive measures, aiming to ensure safe vaccination for the population.

There are still few studies addressing data on IEs from the COVID-19 vaccine^(13)^. The Institute for Safe Medication Practices (ISMP), in collecting voluntary reports of errors or hazards related to the COVID-19 vaccine, identified IEs, including problems related to inadequate dilution of vaccines and even incorrect mixing of products^(18)^. Similar errors may be occurring in different parts of the world, which highlights the need for safe practices in various contexts.

## OBJECTIVES

To analyze the IE related to COVID-19 vaccines in Minas Gerais.

## METHODS

### Ethical aspects

The research was submitted to and approved by the *Universidade Federal de São João del-Rei* Research Ethics Committee. Brazilian standards and guidelines for regulating research involving human beings were met, Resolution CNS 510/2016. Patient consent was waived for this research, as it was conducted with secondary data provided by the State Health Department, ensuring participant anonymity.

### Study design, period and place

This is a descriptive epidemiological study, guided by the Equator network STrengthening the Reporting of OBservational studies in Epidemiology (STROBE), carried out in Minas Gerais, from January to December 2021.

The state of Minas Gerais is made up of 853 municipalities, with an estimated population of 20,539,989 million inhabitants in 2022^(19)^. The state is divided into 14 health macro-regions, namely South, Center-South, Center, Jequitinhonha, West, East, Southeast, North, Northwest, East of the South, Northeast, Triângulo do Sul, Triângulo do Norte and Vale do Aço. The Central macro-region has the largest population, and the smallest is the Jequitinhonha macro-region^(20)^.

### Population or sample; inclusion and exclusion criteria

The database was provided by the Minas Gerais State Health Department and accessed by researchers from the Center for Studies in Assessment and Management in Health and Nursing Services between March and April 2023. The analysis included all records of IE of the COVID-19 vaccine reported in the information system throughout 2021. This period was chosen due to the year in which the vaccine was introduced and the complete availability of data for the year 2021.

### Study protocol

The variables analyzed for IE cases that were made available in the database include: sex (male and female); age group (< 12 years; 12-17; 18-35; 36-49; 50-64; ≥65 years); health macro-region (South; Center-South; Center; Jequitinhonha; West; East; Southeast; North; Northwest; East of the South; Northeast; Triângulo do Sul; Triângulo do Norte; Vale do Aço); type of IE, classified according to the form for reporting/investigating ESAVI associated with the use of vaccine, serum or immunoglobulin (type of immunobiological used, administration errors/administration technique errors, prescription or indication errors, failure to assess contraindications or precautions, expired validity, inadequate interval between doses of proposed regimens, handling errors)^(9)^; COVID-19 vaccines administered (CoronaVac/Butantan COVID-19 (inactivated) adsorbed vaccine, AstraZeneca COVID-19 (recombinant) vaccine, Comirnaty Pfizer COVID-19 (mRNA) vaccine, Janssen COVID-19 (recombinant) vaccine).

For IE analysis, the Brazilian National Immunization Program for COVID-19 vaccines recommendations were used. The vaccines that were available during the study period were: the CoronaVac/Butantan COVID-19 (inactivated) adsorbed vaccine, produced by the laboratory Sinovac Life Sciences Co., Ltd, with a two-dose vaccination schedule with a four-week interval between doses and indicated for use by those over 18 years of age; AstraZeneca COVID-19 (recombinant) vaccine, produced by the Serum Institute of India Pvt. Ltd, Fiocruz/Bio-Manguinhos laboratories and the Covax Facility consortium, indicated for people over 18 years of age with a two-dose vaccination schedule and an eight-week interval between doses; Comirnaty COVID-19 (mRNA) vaccine, produced by the Pfizer/Wyeth laboratory, indicated for use by individuals ≥ 12 years old, with a two-dose vaccination schedule and an eight-week interval between doses; COVID-19 (recombinant) vaccine, produced by the pharmaceutical company Janssen, indicated for those over 18 years old with an initial single-dose vaccination schedule^(4)^, with a booster dose with the same immunobiological recommended from November 2021^(21)^. There was also a recommendation for a booster dose for the other vaccines, using the immunizers AstraZeneca/Fiocruz COVID-19 (recombinant) vaccine, Comirnaty Pfizer/Wyeth COVID-19 (mRNA) vaccine and Janssen COVID-19 (recombinant) vaccine^(4)^.

### Analysis of results, and statistics

A descriptive data analysis was performed, including the frequency distribution and the differences between the proportions, according to demographic characteristics and type of error. The incidence rate (IR) of IE per 100,000 doses administered (DA) was also calculated. For the calculation, the number of IE was considered as the numerator and the number of vaccine doses administered in the period, by health macro-region, as the denominator. The number of doses administered in the period was obtained from the Ministry of Health - *Vacinômetro* COVID-19 website^(22)^. In the state of Minas Gerais, in 2021, 35,089,726 vaccine doses were administered and 2,774 IE were reported. Eight duplicate records and 42 records that were specifically related to ESAVI, without information on IE, were excluded, totaling 2,724 records analyzed.

A database was built with the help of Excel 2023. Epi Info™ was used for data analysis.

## RESULTS

Of the 853 municipalities in the State of Minas Gerais, only 381 (44.6%) reported at least one error related to COVID-19 vaccination in 2021. The overall IE rate was 7.8/100 thousand doses administered.

**Figure 1:**
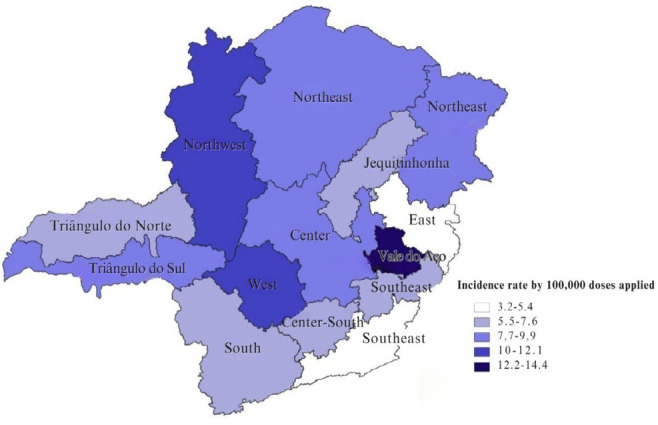
Incidence rate of immunization errors related to COVID-19 vaccines per 100,000 doses administered, health macro-regions, Minas Gerais, Brazil, 2021

Figure 1 shows the incidence of IE by health macro-region of Minas Gerais. The highest incidence of errors was found in the Vale do Aço macro-region, with 14.4/100,000 DA, the West macro-region, with an IR of 11.7/100,000 AD, and the Northwest macro-region, with 10.4/100,000 DA. The health macro-regions with the lowest incidence of reported errors were the Southeast, with 5.0 errors per 100,000 DA, and the East, with 3.2 errors per 100,000 DA.

In relation to the characteristics of the 2,724 reports analyzed, it was observed that females accounted for 1,526 (56.0%) of cases. The most affected age group was ≥ 65 years (24.5%), followed by the age group of 36 - 49 years (23.7%). It was observed that the most frequent error was the type of immunological agent used (37.3%), followed by administration error/technique errors (26.2%). It is noteworthy that 68 (2.5%) individuals under 12 years of age received the COVID-19 vaccine, although in 2021 COVID-19 vaccination was not authorized for this age group with the use of immunobiological agents approved for use at that time (Table 1).

**Table 1:** Characteristics of reports of immunization errors related to COVID-19 vaccines (N = 2,724), Minas Gerais, Brazil, 2021

Variable	n	%
Sex		
Female	1526	56.0
Male	1198	44.0
Age range (years)		
< 12 years	68	2.5
12 to 17 years	451	16.5
18 to 35 years	488	17.9
36 to 49 years	646	23.7
50 to 64 years	404	14.8
≥ 65	667	24.5
Immunization errors		
Type of immunological agent used	1015	37.3
Administration errors/administration technique errors	714	26.2
Prescription or indication errors (outside the recommended age)^(1)^	332	12.2
Inadequate interval between doses of proposed regimens	204	7.5
No assessment of contraindications or precautions	204	7.5
Expired validity	200	7.3
Handling errors	55	2.0

*1) 68 errors were identified in children <12 years old.*

Regarding IR by IE classification, it was observed that the type of immunological agent used was the most incident, 2.9/100 thousand DA, followed by administration errors/technique errors (IR: 2.0/100 thousand DA). Vaccine administered outside the recommended age and no assessment of contraindications or precautions had an incidence of 1.0 and 0.6, respectively, for every 100 thousand DA. The error with the lowest incidence was the handling error, with IR of 0.2/100 thousand DA (data not shown in the table).

Table 2 presents the incidence of IEs by type of COVID-19 vaccine administered. The lowest incidence of errors was observed with Comirnaty Pfizer/Wyeth COVID-19 (mRNA) vaccine, 6.9 errors per 100,000 doses administered.

**Table 2:** Incidence of immunization errors related to COVID-19 vaccines (N = 2,724) by immunobiological agent, Minas Gerais, Brazil, 2021

Immunobiological agent	DA^(1)^	IE^(2)^	IR^(3)^
Janssen COVID-19 (recombinant) vaccine	678,222	82	12.1
Sinovac/Butantan COVID-19 (inactivated) adsorbed vaccine	8,264,747	835	10.1
AstraZeneca/Fiocruz COVID-19 (recombinant) vaccine	12,394,215	861	7.0
Comirnaty Pfizer/Wyeth COVID-19 (mRNA) vaccine	13,752,542	946	6.9

*1) DA: number of records of doses applied; 2) IE: number of records of immunization errors; 3) IR: incidence rate of immunization errors per 100 thousand doses applied.*

## DISCUSSION

In Minas Gerais, heterogeneity of IEs against COVID-19 and possible underreporting of these errors were observed. The estimated incidence was 7.8 IEs for the state, with the Vale do Aço, Oeste and Noroeste macro-regions having the highest incidence. The type of immunobiological agent and administration/technique errors were the most frequent errors, and Comirnaty Pfizer/Wyeth COVID-19 (mRNA) vaccine had the lowest incidence of errors.

According to the epidemiological bulletin for monitoring the safety of COVID-19 vaccines, since the beginning of the campaign until epidemiological week 11/2023, 200,863 reports were documented in the *e-SUS Notifica* system. Of these reports, 23% (46,147) were related to IE^(23)^. If we calculate the incidence of IE based on DA in the 26-month period (384,827,394), we obtain an incidence of 12 IE per 100,000 DA. The data from our study include the beginning of the approval and implementation of new vaccines, which generated logistical and operational challenges and may have led to an increase in the incidence of these errors, which justifies the difference with incidence in Brazil published in the bulletin^(23)^.

Vaccination is one of the main health actions carried out in Primary Health Care (PHC). In 2017, patient safety became part of the Brazilian National Primary Care Policy (In Portuguese, *Política Nacional de Atenção Básica* - PNAB), promoting safe care and fostering a culture of safety among PHC professionals^(24)^. With the introduction of patient safety as a formal guideline within PNAB, there was a push to implement practices that minimize risks and errors that could negatively affect patients.

One of the hallmarks of a patient safety culture is the promotion of a culture that encourages and rewards the identification, reporting, and resolution of safety-related issues^(11)^. In this context, it is clear that error reporting systems are considered essential safety mechanisms and contribute to a positive organizational culture and provision of high-quality care^(25)^.

In Brazil, the completion of information on IE is integrated into the same information system used for reporting ESAVI, considering the possibility of adverse events after an error occurs. Therefore, health surveillance plays an important role in identifying IE through ESAVI reporting, especially during vaccination campaigns, when there is an increase in available human and physical resources^(16)^.

The results of this study point to a possible underreporting of IE, supporting findings from other studies carried out in Minas Gerais^(10,26)^ and Paraná^(27)^ and even internationally^(28)^. In Verona, Italy, a study to identify errors in the COVID-19 vaccination process attributes the low number of errors and near misses reported to underreporting, which cannot always be effectively controlled^(29)^. Furthermore, the requirement and responsibility of vaccination activity were also mentioned as contributing factors^(29)^.

Despite the importance of reporting, incidents are often underreported. This is often related to feelings of fear of punishment, guilt and shame. This dynamic perpetuates the tendency to omit such episodes, resulting in the loss of the opportunity to learn about them and treat them appropriately^(30,31)^. The punitive environment needs to be replaced by a culture of knowledge exchange, in which healthcare professionals can discuss failures without fear^(25)^.

Even with significant progress in patient safety, human error still stands out as a critical factor. Incidents involving errors by healthcare professionals often gain prominence in the press and media, provoking intense and negative public reaction^(25)^. There have been several reports in the media about errors in COVID-19 vaccination, which vary in nature and severity^(32,33)^. Media coverage can create an environment where healthcare professionals feel afraid to report, especially if these errors are portrayed in a sensationalist or punitive manner, which can result in underreporting.

The results also showed that a higher incidence of errors was found in the Vale do Aço macro-region, which does not necessarily mean a higher occurrence of the event when we consider the possible presence of underreporting. The authors assume that this finding is related to greater reporting, possibly related to an organizational culture focused on patient safety^(26)^. Errors are usually more explicit in institutions that have a more solid safety culture, as healthcare professionals working in these environments feel more comfortable reporting adverse events^(34)^, including IE.

In addition to being a safety mechanism, reporting IE should contribute to an environment of continuous learning. If the reported data is simply archived, without proper treatment for improvement actions and discussion with all professionals in the institution, there will be no organizational learning. To develop a strengthened safety culture, leadership must use this data in care management, promoting training and discussion about reported errors^(25)^.

It was observed that the most frequent IE was the type of immunological agent used, followed by administration error/technique errors. A study carried out in Italy also identified a higher proportion of errors related to vaccine administration, whether in principle, dosage or recommended interval^(29)^. It is important to highlight that this term, in Italy, also included the error related to the type of immunobiological agent used, corroborating the findings of our study.

The availability of four COVID-19 vaccines, with specific requirements in terms of dosage, interval between doses and storage conditions, in addition to being targeted to specific population groups based on characteristics such as age, underlying medical conditions or occupation^(4)^, may have contributed to the higher incidence of these types of errors identified in the study. In this regard, clinical screening is important for patient safety in the vaccination process, and users’ analysis needs to include aspects such as recommendation, contraindication, health history and vaccination status^(35)^.

COVID-19 vaccine administration to children under 12 years of age before authorization by ANVISA^(4)^ deserves special attention, as it implies significant risks to the safety and efficacy of vaccination. This practice may include a misinterpretation of guidelines or a lack of understanding of the specific characteristics of each vaccine, its dosing schedules and special requirements. Moreover, familiarization with new vaccines, protocols and processes can take some time, and the pressure to vaccinate the population quickly has contributed to an increased risk of errors. In the United States, there were 14.7% of reports of vaccine administration to unauthorized age groups^(13)^. The modification of an internal and external context that culminates in an increase in work overload and pressure requires an adaptation in planning and work team reallocation, being achieved with knowledge of the responsibilities of the professionals involved^(25)^.

Comirnaty Pfizer/Wyeth COVID-19 (mRNA) vaccine had the lowest incidence of errors. We can assume that the particularities of this vaccine, in relation to specific storage and handling conditions, reconstitution and dosages, may have resulted in more careful and accurate administration. Furthermore, healthcare professionals may have received additional training to ensure accuracy in these processes, given the sensitivity of the vaccine to these aspects.

### Study limitations

The limitations of this study are related to the use of secondary data, which does not allow controlling IE underreporting or data quality, which may underestimate the real occurrence of the error.

### Contributions to health, nursing or public policy

The results of this study can guide actions and strategies for preventing IE by managers and professionals with the aim of ensuring patient safety and reducing the incidence of these errors in vaccination rooms. The identification of possible underreporting highlights the need to discuss initiatives so that professionals feel encouraged to report errors without fear of punishment, contributing to an environment of continuous learning and safe vaccination.

## CONCLUSIONS

Reports of IE during COVID-19 vaccination in Minas Gerais point to the heterogeneity in incidence rates across the state’s various macro-regions and to possible underreporting of these errors.

Investments in the management of immunization services, professional qualification, standardization of procedures and discussion of reported errors are priority strategies to minimize the occurrence of IE and establish a culture of safety in the vaccination room.

The experience gained during the vaccination campaign in response to the COVID-19 pandemic will certainly provide insights to improve and organize future mass vaccination campaigns, with the aim of ensuring safe vaccination for the entire population.

## Data Availability

https://doi.org/10.17632/hj7k98t6pn.1
