# Supplementary material for: Immunization errors of COVID-19 vaccines in Minas Gerais
Source: Rev Bras Enferm. 2025 Aug 8;78(3):e20230525. doi: 10.1590/0034-7167-2023-0525 (PMC12334198; doi:10.1590/0034-7167-2023-0525)
Supplement: Translation

# Translation: Erros de imunização das vacinas contra COVID-19 em Minas Gerais

**DOI:** 10.1590/0034-7167-2023-0525pt

**Authors:** Wiara Viana Ferreira, Thayane Ingrid Xavier de Andrade, Thays Cristina Pereira Barbosa, Rillary Carvalho dos Santos, Stênio Henrique Oliveira, Vinícius Carvalho Guimarães, Eliete Albano de Azevedo Guimarães, Valeria Conceição de Oliveira

**Affiliations:** I Universidade Federal de São João Del Rei. Divinópolis, Minas Gerais, Brasil; II Universidade de São Paulo. Ribeirão Preto, São Paulo, Brasil

**Keywords:** Vacinas contra COVID-19, Erros de Medicação, Programas de Imunização, Vacinação em Massa, Epidemiologia Descritiva.

## Abstract

**Objetivos:**

analisar os erros de imunização relacionados às vacinas contra COVID-19 em Minas Gerais.

**Métodos:**

estudo epidemiológico descritivo, realizado em Minas Gerais com dados do e-SUS Notifica no ano de 2021. Foram realizados análise descritiva e cálculo da taxa de incidência por macrorregiões de saúde do estado.

**Resultados:**

dos 853 municípios, apenas 381 (44,7%) notificaram algum erro de imunização, totalizando 2.724 notificações. A incidência dos erros notificados no estado foi de 7,8/100 mil doses aplicadas, sendo a macrorregião do Vale do Aço (14,4/100 mil) a de maior incidência. O erro mais frequente foi o tipo de imunológico utilizado (37,3%), e a vacina da Pfizer teve a menor incidência de erro.

**Conclusões:**

os erros de imunização da vacina contra COVID-19 notificados em Minas Gerais apontam para a heterogeneidade nas taxas de incidência das diversas macrorregionais do estado, além da subnotificação desses erros.

## INTRODUÇÃO

Em março de 2020, a Organização Mundial da Saúde declarou como pandemia o surto do novo coronavírus (SARS-CoV-2)^(1)^ e, durante esse ano, a população acompanhou de perto os esforços da comunidade científica no desenvolvimento das etapas dos estudos de vacinas contra o SARS-CoV-2^(2)^. Em 17 de janeiro de 2021, a Agência Nacional de Vigilância Sanitária (ANVISA) concedeu a autorização para o uso emergencial da vacina adsorvida COVID-19 (inativada) Sinovac/Butantan e vacina COVID-19 (recombinante) AstraZeneca/Fiocruz^(2)^.

Para coordenar a campanha de vacinação no Brasil, criou-se o Plano Nacional de Operacionalização da Vacinação contra COVID-19 com o objetivo de estruturar a distribuição das vacinas e definir grupos prioritários^(3)^. Durante o ano de 2021, as vacinas aprovadas e introduzidas foram vacina adsorvida COVID-19 (inativada) CoronaVac/Butantan, vacina COVID-19 (recombinante) AstraZeneca, vacina COVID-19 (RNAm) Comirnaty Pfizer e vacina COVID-19 (recombinante) Janssen, o que permitiu ampliar a vacinação para além dos grupos prioritários e acelerar o processo de vacinação da população^(3,4)^.

Diante da necessidade de ampliar e agilizar a vacinação contra COVID-19, foram implementadas várias estratégias, como a criação de *drive-thrus*, a contratação de mais profissionais de saúde para administração de vacinas, a capacitação contínua desses profissionais e a oferta de vacinação domiciliar^(5,6)^. Essas medidas visavam garantir que o maior número de pessoas fosse vacinado de maneira rápida e eficiente, porém criaram riscos potenciais para a segurança do paciente devido à possibilidade de erros de imunização (EI).

Os EI não são simplesmente resultados de falhas individuais, mas resultados da interação entre os indivíduos e os sistemas nos quais estão inseridos^(7)^, frequentemente sendo o produto de uma dinâmica complexa entre diferentes fatores. Esses fatores podem incluir cultura organizacional, sistemas e processos, falta de recursos, comunicação inadequada, estresse no trabalho, treinamento insuficiente, entre outros^(8)^. Os erros são inerentes a qualquer ação humana, independentemente do campo de atividade, refletindo uma característica constante do processo cognitivo humano^(7)^.

O EI pode ser conceituado como qualquer evento evitável devido ao uso incorreto dos imunobiológicos, procedimentos e sistemas que podem causar redução ou ausência de imunidade e evento supostamente atribuível à vacinação ou imunização (ESAVI) grave^(9)^.

Ao analisarmos todos os fatores que podem levar ao EI, percebemos que o profissional que o desencadeou está na etapa final da cadeia, mas talvez não seja a origem do problema. Se for possível intervir nesse estágio, por meio de ações preventivas, é possível evitar futuros erros^(7)^.

A equipe de enfermagem atua como uma barreira final na prevenção de EI^(10)^. No entanto, o processo de vacinação é complexo e abrange várias etapas, incluindo produção, logística de distribuição, armazenamento, gestão e comunicação, cada uma essencial ao sucesso da vacinação. Assumir a responsabilidade compartilhada pela segurança do paciente é crucial para o desenvolvimento e manutenção de sistemas de saúde seguros e eficientes^(11)^.

A literatura já vem apontando que a introdução de novos imunobiológicos é um fator contributivo para o aumento do EI^(12-15)^. Ademais, em situações de grande escala, como a campanha de vacinação contra COVID-19, pode ocorrer um aumento na incidência de erros e ESAVI^(16,17)^. Por isso, é importante o monitoramento da ocorrência do erro para conhecimento e elucidação de suas causas e efeitos, além da implantação de medidas preventivas, visando garantir uma vacinação segura à população.

Ainda são escassos os trabalhos que abordam dados sobre os EI da vacina contra COVID-19^(13)^. O *Institute for Safe Medication Practices* (ISMP), na coleta de relatórios voluntários de erros ou perigos relacionados à vacina da COVID-19, identificou EI, incluindo problemas relacionados à diluição inadequada de vacinas até misturas equivocadas de produtos^(18)^. Erros semelhantes podem estar ocorrendo em diferentes partes do mundo, o que sublinha a necessidade de práticas seguras em vários contextos.

## OBJETIVOS

Analisar os EI relacionados às vacinas contra COVID-19 em Minas Gerais.

## MÉTODOS

### Aspectos éticos

A pesquisa foi submetida e aprovada pelo Comitê de Ética em Pesquisa da Universidade Federal de São João del-Rei. Foram atendidas as normas e diretrizes brasileiras de regulamentação de pesquisas envolvendo seres humanos, Resolução CNS 510/2016. O consentimento do paciente foi dispensado para esta pesquisa, pois ela foi realizada com dados secundários disponibilizados pela Secretaria de Saúde do Estado, garantindo-se o anonimato dos participantes.

### Desenho, período e local do estudo

Trata-se de estudo epidemiológico descritivo, norteado pela ferramenta *STrengthening the Reporting of OBservational studies in Epidemiology* (STROBE) da rede Equator, realizado em Minas Gerais, no período de janeiro a dezembro de 2021.

O estado de Minas Gerais é composto por 853 municípios, com população estimada de 20.539.989 milhões de habitantes no ano de 2022^(19)^. O estado é dividido em 14 macrorregiões de saúde, sendo elas Sul, Centro-Sul, Centro, Jequitinhonha, Oeste, Leste, Sudeste, Norte, Noroeste, Leste do Sul, Nordeste, Triângulo do Sul, Triângulo do Norte e Vale do Aço. A macrorregião Centro é a de maior porte populacional, e a de menor porte é a macrorregião de Jequitinhonha^(20)^.

### População ou amostra; critérios de inclusão e exclusão

A base de dados foi fornecida pela Secretaria de Estado de Saúde de Minas Gerais e acessada por pesquisadores do Núcleo de Estudos em Avaliação e Gestão em Serviço de Saúde e Enfermagem entre março e abril de 2023. A análise englobou todos os registros de EI da vacina contra COVID-19 notificados no sistema de informação ao longo de 2021. A escolha deste período se deve ao ano em que a vacina foi introduzida e à completa disponibilidade dos dados referentes ao ano de 2021.

### Protocolo do estudo

As variáveis analisadas para os casos de EI que foram disponibilizadas no banco de dados incluem: sexo (masculino e feminino); faixa etária (< 12 anos; 12-17; 18-35; 36-49; 50-64; ≥65 anos); macrorregião de saúde (Sul; Centro-Sul; Centro; Jequitinhonha; Oeste; Leste; Sudeste; Norte; Noroeste; Leste do Sul; Nordeste; Triângulo do Sul; Triângulo do Norte; Vale do Aço); tipo de EI, classificado segundo o formulário para notificação/investigação de ESAVI associado ao uso de vacina, soro ou imunoglobulina (tipo de imunobiológico utilizado, erros de administração/erros de técnica de administração, erros de prescrição ou indicações, não avaliação de contraindicação ou precauções, validade vencida, intervalo inadequado entre dose dos esquemas propostos, erros de manuseio)^(9)^; vacinas de COVID-19 administradas (vacina adsorvida COVID-19 (inativada) CoronaVac/Butantan, vacina COVID-19 (recombinante) AstraZeneca, vacina COVID-19 (RNAm) Comirnaty Pfizer, vacina COVID-19 (recombinante) Janssen).

Para análise dos EI, utilizaram-se as recomendações do Programa Nacional de Imunização para as vacinas contra COVID-19. As vacinas que estavam disponíveis no período do estudo foram: a vacina adsorvida COVID-19 (inativada) CoronaVac/Butantan, produzida pelo laboratório *Sinovac Life Sciences Co*., Ltd, tendo esquema vacinal de duas doses com intervalo de quatro semanas entre as doses e indicação de uso para maiores de 18 anos; vacina COVID-19 (recombinante) AstraZeneca, produzida pelos laboratórios *Serum Institute of India Pvt*. Ltd, Fiocruz/Bio-Manguinhos e consórcio *Covax Facility*, indicada para maiores de 18 anos com esquema vacinal de duas doses e intervalo de oito semanas entre doses; vacina COVID-19 (RNAm) (Comirnaty), produzida pelo laboratório Pfizer/Wyeth, tendo indicação de uso para indivíduos ≥ 12 anos, esquema vacinal de duas doses e intervalo de oito semanas entre doses; vacina COVID-19 (recombinante), produzida pela farmacêutica Janssen, indicada para maiores de 18 anos com esquema vacinal inicial de dose única^(4)^, sendo recomendada, a partir de novembro de 2021, uma dose de reforço com o mesmo imunobiológico^(21)^. Houve também a recomendação de uma dose de reforço para as demais vacinas, utilizando os imunizantes vacina COVID-19 (recombinante) AstraZeneca/Fiocruz, vacina COVID-19 (RNAm) (Comirnaty) Pfizer/Wyeth e vacina COVID-19 (recombinante) Janssen^(4)^.

### Análise dos resultados e estatística

Foi realizada análise descritiva dos dados, incluindo a distribuição de frequências e as diferenças entre as proporções, segundo características demográficas e tipo de erro. Calculou-se ainda a taxa de incidência (TI) dos EI por 100 mil doses aplicadas (DA). Para o cálculo, foi considerado o número de EI como numerador e o número de doses de vacinas administradas no período, por macrorregião de saúde, como denominador. O número de doses administradas no período foi obtido no site do Ministério da Saúde - Vacinômetro COVID-19^(22)^. No estado de Minas Gerais, no período de 2021, foram administradas 35.089.726 doses de vacinas e notificados 2.774 EI. Foram excluídos 8 registros duplicados e 42 registros que se relacionavam especificamente a ESAVI, sem informações sobre o EI, totalizando 2.724 registros analisados.

Foi construído um banco de dados com o auxílio do *software* Excel 2023. Utilizou-se o *Software* Epi Info™ para a análise dos dados.

## RESULTADOS

Dos 853 municípios do estado de Minas Gerais, apenas 381 (44,6%) realizaram a notificação pelo menos um erro relacionado à vacinação contra COVID-19 em 2021. A TI geral de EI foi de 7,8/100 mil DA.

A Figura 1 apresenta a incidência dos EI por macrorregião de saúde de Minas Gerais. Foram encontradas maiores incidências de erros na macrorregião do Vale do Aço, com 14,4/100 mil DA, macrorregião Oeste, com TI de 11,7/100 mil DA, e macrorregião Noroeste, com 10,4/100 mil DA. Já as macrorregiões de saúde com menores incidências de erros notificados foram a Sudeste, com 5,0 erros por 100 mil DA, e a Leste, com 3,2 erros por 100 mil DA.

**Figura 1:** Taxa de incidência de erros de imunização relacionados às vacinas contra COVID-19 por 100 mil doses aplicadas, macrorregiões de saúde, Minas Gerais, Brasil, 2021

Em relação às características das 2.724 notificações analisadas, observou-se que o sexo feminino representou 1.526 (56,0%) dos casos. A faixa etária mais acometida foi a de ≥ 65 anos (24,5%), seguida da faixa etária de 36 - 49 anos (23,7%). Observou-se que o erro mais frequente foi o tipo de imunológico utilizado (37,3%), seguido do erro de administração/erros de técnica (26,2%). Destaca-se que 68 (2,5%) indivíduos menores de 12 anos receberam a vacina de COVID-19, embora que, no ano de 2021, a vacinação contra COVID-19 não estivesse autorizada para essa faixa etária com a utilização dos imunobiológicos aprovados para uso naquele momento (Tabela 1).

**Tabela 1:** Características das notificações dos erros de imunização relacionados às vacinas contra COVID-19 (N = 2.724), Minas Gerais, Brasil, 2021

Variável	n	%
Sexo		
Feminino	1526	56,0
Masculino	1198	44,0
Faixa etária (em anos)		
< 12 anos	68	2,5
12 a 17 anos	451	16,5
18 a 35 anos	488	17,9
36 a 49 anos	646	23,7
50 a 64 anos	404	14,8
≥ 65	667	24,5
Erros de imunização		
Tipo de imunológico utilizado	1015	37,3
Erros de administração/erros de técnica de administração	714	26,2
Erros de prescrição ou indicação (fora da idade recomendada)^(1)^	332	12,2
Intervalo inadequado entre dose dos esquemas propostos	204	7,5
Não avaliação de contraindicações ou precauções	204	7,5
Validade vencida	200	7,3
Erros de manuseio	55	2,0

*1) Foram identificados 68 erros em crianças <12 anos.*

Em relação à TI por classificação do EI, observou-se que o tipo de imunológico utilizado foi o mais incidente (2,9/100 mil DA), seguido de erros de administração/erros de técnica (TI: 2,0/100 mil DA). Vacina administrada fora da idade recomendada e não avaliação de contraindicações ou precauções tiveram incidência de 1,0 e 0,6, respectivamente, a cada 100 mil DA. O erro com menor incidência foi o erro de manuseio, com TI de 0,2/100 mil DA (dados não apresentados em tabela).

A Tabela 2 apresenta a incidência dos EI por tipo de vacina de COVID-19 administrada. A menor incidência de erros foi observada na vacina COVID-19 (RNAm) (Comirnaty) Pfizer/Wyeth, com 6,9 erros para cada 100 mil doses administradas.

**Tabela 2:** Incidência dos erros de imunização relacionados às vacinas contra COVID-19 (N = 2.724) por imunobiológico, Minas Gerais, Brasil, 2021

Imunobiológico	DA^(1)^	EI^(2)^	TI^(3)^
Vacina COVID-19 (recombinante) Janssen	678.222	82	12,1
Vacina adsorvida COVID-19 (inativada) Sinovac/Butantan	8.264.747	835	10,1
Vacina COVID-19 (recombinante) AstraZeneca/Fiocruz	12.394.215	861	7,0
Vacina COVID-19 (RNAm) (Comirnaty) Pfizer/Wyeth	13.752.542	946	6,9

*1) DA: número de registros de doses aplicadas; 2) EI: número de registros de erros de imunização; 3) TI: taxa de incidência de erros de imunização por 100 mil doses aplicadas.*

## DISCUSSÃO

Em Minas Gerais, observaram-se heterogeneidade das TI de EI contra COVID-19 e possível subnotificação desses erros. A incidência estimada foi de 7,8 EI para o estado, com as macrorregiões Vale do Aço, Oeste e Noroeste sendo as de maior incidência. O tipo de imunobiológico e a administração/erros de técnica foram os erros mais frequentes, e a vacina COVID-19 (RNAm) (Comirnaty) Pfizer/Wyeth teve a menor incidência de erro.

Segundo o boletim epidemiológico de monitoramento da segurança das vacinas contra COVID-19, desde o início da campanha até a semana epidemiológica 11/2023, foram documentadas 200.863 notificações no sistema e-SUS Notifica. Dessas notificações, 23% (46.147) estavam relacionadas a EI^(23)^. Se calcularmos a incidência de EI com base nas DA no período de 26 meses (384.827.394), obteremos uma incidência de 12 EI por 100 mil DA. Os dados do nosso estudo incluem o início da aprovação e implementação das novas vacinas, o que gerou desafios logísticos e operacionais, e podem ter propiciado o aumento na incidência desses erros, o que justifica a diferença com a incidência no Brasil publicada no boletim^(23)^.

A vacinação é uma das principais ações de saúde realizadas na Atenção Primária à Saúde (APS). Em 2017, a segurança do paciente passou a fazer parte da Política Nacional de Atenção Básica (PNAB), promovendo cuidados seguros e fomentando uma cultura de segurança entre os profissionais da APS^(24)^. Com a introdução da segurança do paciente como uma diretriz formal dentro da PNAB, houve um impulso para implementar práticas que minimizem riscos e erros que poderiam afetar negativamente os pacientes.

Uma das características da cultura de segurança do paciente é a promoção de uma cultura que incentiva e recompensa a identificação, notificação e resolução de problemas relacionados à segurança^(11)^. Nesse contexto, é evidente que os sistemas de notificação de erros são considerados mecanismos essenciais de segurança e contribuem para uma cultura organizacional positiva e para a prestação de uma assistência de alta qualidade^(25)^.

No Brasil, o preenchimento das informações sobre o EI está integrado ao mesmo sistema de informação usado para a notificação de ESAVI, considerando a possibilidade de eventos adversos após a ocorrência de um erro. Portanto, a vigilância em saúde desempenha um papel importante na identificação dos EI através das notificações de ESAVI, especialmente durante as campanhas de vacinação, quando há um aumento nos recursos humanos e físicos disponíveis^(16)^.

Os resultados deste estudo apontam para uma possível subnotificação de EI, corroborando os achados de outros estudos realizados em Minas Gerais^(10,26)^ e Paraná^(27)^ e até internacionalmente^(28)^. Em Verona, na Itália, estudo para identificar erros no processo de vacinação da COVID-19 atribui o baixo número de erros e quase acidentes notificados à subnotificação, que nem sempre pode ser eficazmente controlada^(29)^. Além disso, a exigência e a responsabilidade da atividade de vacinação também foram mencionadas como fatores contribuintes^(29)^.

Apesar da relevância da notificação, as ocorrências frequentemente são subnotificadas. Isso geralmente está relacionado a sentimentos de medo de punições, culpa e vergonha. Essa dinâmica perpetua a tendência à omissão de tais episódios, resultando na perda da oportunidade de conhecê-los e tratá-los de maneira adequada^(30,31)^. O ambiente punitivo precisa ser substituído por uma cultura de troca de conhecimento, em que os profissionais de saúde possam discutir as falhas sem receio^(25)^.

Mesmo com progressos significativos na segurança do paciente, o erro humano ainda se destaca como um fator crítico. Frequentemente, incidentes envolvendo erros de profissionais de saúde ganham destaque na imprensa e nos meios de comunicação, provocando intensa e negativa reação pública^(25)^. Foram vários relatos reportados à mídia sobre erros na vacinação contra COVID- 19, que variam em natureza e gravidade^(32,33)^. A midiatização pode criar um ambiente onde os profissionais de saúde sintam receio de notificar, principalmente, se esses erros forem retratados de maneira sensacionalista ou punitiva, o que pode resultar em subnotificação.

Os resultados ainda mostraram que foi encontrada maior incidência de erro na macrorregião do Vale do Aço, o que não significa, necessariamente, maior ocorrência do evento quando consideramos a possível presença da subnotificação. Os autores pressupõem que esse achado esteja relacionado a uma maior notificação, possivelmente relacionada a uma cultura organizacional voltada à segurança do paciente^(26)^. Habitualmente, os erros são mais explícitos em instituições que possuem uma cultura de segurança mais sólida, pois profissionais de saúde inseridos nesses ambientes se sentem mais confortáveis em relatar eventos adversos^(34)^, incluindo EI.

A notificação de EI, além de ser um mecanismo de segurança, deve contribuir para um ambiente de aprendizado contínuo. Se os dados notificados forem simplesmente arquivados, sem o devido tratamento para ações de melhoria e discussão com todos os profissionais da instituição, não haverá aprendizado organizacional. Para desenvolver uma cultura de segurança fortalecida, a liderança deve utilizar esses dados na gestão dos cuidados, promovendo a capacitação e a discussão sobre os erros notificados^(25)^.

Observou-se que o EI mais frequente foi o tipo de imunológico utilizado, seguido do erro de administração/erros de técnica. Estudo realizado na Itália também identificou maior proporção de erros relacionados à administração da vacina, seja em princípio, dosagem ou intervalo recomendado^(29)^. Importante ressaltar que esse termo, na Itália, incluiu também o erro relacionado ao tipo de imunobiológico utilizado, corroborando os achados do nosso estudo.

A disponibilidade de quatro vacinas contra COVID-19, com requisitos específicos em termos de dosagem, intervalo entre as doses e condições de armazenamento, além de ser direcionadas a grupos populacionais específicos com base em características como idade, condições médicas subjacentes ou profissão^(4)^, podem ter contribuído para a maior incidência desses tipos de erros identificados no estudo. Nesse sentido, a triagem clínica é importante para a segurança do paciente no processo de vacinação, sendo que a análise do usuário precisa envolver aspectos como recomendação, contraindicação, histórico de saúde e situação vacinal^(35)^.

A administração da vacina contra COVID-19 em menores de 12 anos antes da autorização da ANVISA^(4)^ merece uma atenção especial, por implicar riscos significativos para a segurança e eficácia da vacinação. Essa prática pode incluir uma interpretação equivocada das diretrizes ou falta de compreensão sobre as características específicas de cada vacina, seus esquemas de dosagem e requisitos especiais. Ademais, a familiarização com novas vacinas, protocolos e processos pode levar algum tempo, e a pressão para vacinar rapidamente a população contribuiu para aumentar o risco de erros. Nos Estados Unidos, houve 14,7% de relatos de administração de vacinas a grupos etários não autorizados^(13)^. A modificação de um contexto interno e externo que culmina em um aumento da sobrecarga de trabalho e pressão necessita de uma adaptação no planejamento e uma realocação da equipe de trabalho, sendo alcançado com o conhecimento das responsabilidades dos profissionais envolvidos^(25)^.

A vacina COVID-19 (RNAm) (Comirnaty) Pfizer/Wyeth foi a de menor incidência de erro. Podemos pressupor que as particularidades dessa vacina, em relação às condições específicas de armazenamento e manuseio, reconstituição e dosagens, podem ter resultado em uma administração mais cuidadosa e precisa. Ademais, os profissionais de saúde podem ter recebido capacitação adicional para garantir a precisão nesses processos, devido à sensibilidade da vacina a esses aspectos.

### Limitações do estudo

As limitações deste estudo estão relacionadas à utilização de dados secundários, que não possibilitam controlar a subnotificação de EI tampouco a qualidade do dado, o que pode subestimar a real ocorrência do erro.

### Contribuições para as áreas da saúde, enfermagem, ou políticas públicas

Os resultados deste estudo podem nortear ações e estratégias de prevenção ao EI pelos gestores e profissionais com o objetivo de garantir a segurança do paciente e reduzir a incidência desses erros nas salas de vacinação. A identificação de uma possível subnotificação destaca a necessidade de discutir iniciativas para que os profissionais se sintam encorajados a notificar os erros sem medo de punição, contribuindo para um ambiente de aprendizado contínuo e da vacinação segura.

## CONCLUSÕES

As notificações de EI durante a vacinação contra COVID-19 em Minas Gerais apontam para a heterogeneidade nas taxas de incidência das diversas macrorregionais do estado e para uma possível subnotificação desses erros.

Investimentos na gestão de serviços de imunização, na qualificação profissional, na padronização de procedimentos e na discussão dos erros notificados são estratégias prioritárias para minimizar a ocorrência dos EI e estabelecer a cultura de segurança em sala de vacinação.

A experiência adquirida durante a campanha de vacinação diante da pandemia de COVID-19 certamente fornecerá *insights* para aprimorar e organizar futuras campanhas de vacinação em massa, com o objetivo de garantir uma vacinação segura para toda a população.

## Data Availability

https://doi.org/10.17632/hj7k98t6pn.1
